# Implementing a chest X-ray artificial intelligence tool to enhance tuberculosis screening in India: Lessons learned

**DOI:** 10.1371/journal.pdig.0000404

**Published:** 2023-12-07

**Authors:** Shibu Vijayan, Vaishnavi Jondhale, Tripti Pande, Amera Khan, Miranda Brouwer, Asha Hegde, Ravdeep Gandhi, Venkatesh Roddawar, Shilpa Jichkar, Aniruddha Kadu, Sandeep Bharaswadkar, Mayank Sharma, Nathaly Aguilera Vasquez, Lucky Richardson, Dennis Robert, Saniya Pawar

**Affiliations:** 1 Qure.ai, Bangalore, Karnataka, India; 2 PATH, Mumbai, India; 3 External consultant, Washington DC, United States of America; 4 STOP TB Partnership, Geneva, Switzerland; 5 PHTB Consult, 9731 AK. Groningen, The Netherlands; 6 John Snow, Inc., New Delhi, Delhi, India; 7 Department of Health Services, Nagpur Municipal Corporation, Nagpur, India; 8 World Health Organization, India; 9 Bill and Melinda Gates Foundation, India; 10 McGill International TB Centre, Montreal, Québec, Canada; 11 Disha Foundation, Nagpur, India; Indiana University Purdue University at Indianapolis, UNITED STATES

## Abstract

Artificial Intelligence (AI) based chest X-ray (CXR) screening for tuberculosis (TB) is becoming increasingly popular. Still, deploying such AI tools can be challenging due to multiple real-life barriers like software installation, workflow integration, network connectivity constraints, limited human resources available to interpret findings, etc. To understand these challenges, PATH implemented a TB REACH active case-finding program in a resource-limited setting of Nagpur in India, where an AI software device (qXR) intended for TB screening using CXR images was used. Eight private CXR laboratories that fulfilled prerequisites for AI software installation were engaged for this program. Key lessons about operational feasibility and accessibility, along with the strategies adopted to overcome these challenges, were learned during this program. This program also helped to screen 10,481 presumptive TB individuals using informal providers based on clinical history. Among them, 2,303 individuals were flagged as presumptive for TB by a radiologist or by AI based on their CXR interpretation. Approximately 15.8% increase in overall TB yield could be attributed to the presence of AI alone because these additional cases were not deemed presumptive for TB by radiologists, but AI was able to identify them. Successful implementation of AI tools like qXR in resource-limited settings in India will require solving real-life implementation challenges for seamless deployment and workflow integration.

## Introduction

An estimated 10.6 million individuals were affected by tuberculosis (TB) worldwide in 2021, and 28% were from India alone [1]. The COVID-19 pandemic has adversely affected TB notifications (TB notifications refer to newly detected cases of TB) since 2020. India contributed to 41% of the global decline in TB notifications, which was the most by any country [2]. In 2019, about 2.4 million cases were reported in India, and it is estimated that approximately 540,000 cases got ‘missed’ and were never reported [3]. Besides, with an ambitious target of eliminating TB by 2025 [4], India has to scale and implement sustainable initiatives across its states to tackle the burden of TB.

Maharashtra, India’s second most populous state, has reported the second-highest TB burden, contributing to about 9% of the notified TB cases in 2019 [3]. Nationally, the private sector represents only 28% of the notifications of individuals with TB. Yet, it is the first point of contact for over 80% of individuals seeking health care and manages over 50% of individuals with TB [3,5]. It is thus evident that engaging the private sector is very important to achieving India’s ambitious TB elimination target.

Chest X-rays (CXR) have been identified as a screening and triaging tool for TB disease. In the TB diagnostic algorithm in India, the National TB Elimination Program (NTEP) recommends CXR for all individuals in conjunction with sputum smear examination [6]. A study evaluating diagnostic and treatment practices among private-sector physicians in Chennai noted that CXR was a preferred screening tool amongst them [7]. However, implementing CXR for TB screening is often challenging in resource-limited settings due to a lack of qualified individuals such as radiologists for interpretation, inter- and intra-reader variability, and limited infrastructure for CXR facilities [8,9]. Leveraging novel digital health technological advances, such as artificial intelligence (AI) and image recognition, can support healthcare professionals in screening diseases, including TB. These technologies are being tested in various countries worldwide [10].

One such AI-based software for CXR interpretation assistance is qXR, developed by Qure.ai, an AI company developing AI software for healthcare. qXR has received CE marking, a European quality certification, to detect TB abnormalities in adults over 15 years [11]. qXR analyzes CXR images and notifies healthcare professionals in case of the presence or absence of radiological signs of TB, thereby aiding in downstream patient diagnosis and management.

Previous studies have evaluated the diagnostic accuracy of qXR against human readers, using Xpert MTB/RIF [12] as a reference standard. In a study conducted in India, qXR showed moderate specificity and sensitivity for detecting pulmonary TB [13]. Another study conducted using CXRs from Nepal and Cameroon showed higher specificity in comparison to radiologists while maintaining similar sensitivity [14]. An evaluation of multiple AI algorithms intended for detecting radiological signs of TB in CXRs using data from patients in a high tuberculosis burden setting in Bangladesh reported that qXR has an AUC (area under the curve) of 90·81% and that it met the WHO’s Target Product Profile criteria of minimum 70% specificity at 90% sensitivity [15,16]. Another independent evaluation of 12 different AI algorithms for TB detection conducted in Vietnam found an AUC of about 82% for qXR [17]. While these studies evaluated the diagnostic accuracy, none reported the experience of implementing an AI software device in a primary care setting. Further to our knowledge, no current literature illustrates lessons learned on implementing such a technology in a private primary care setting. As interest is growing in using digital health technologies to facilitate care, there is a need to understand how to best introduce and implement these tools in new settings. Implementation frameworks allow understanding innovative health interventions’ operational feasibility and acceptability [18,19]. Addressing the real-life implementation challenges and devising a strategy to help tackle the challenges associated with integrating AI solutions into care settings is critical.

In this article, the implementational challenges faced by PATH [20] during the AI software device implementation in a resource-limited setting of Nagpur, Maharashtra, and the strategies adopted by PATH to address these barriers are discussed. Since 2014, PATH has been instrumental in empowering private sector engagement initiatives in India, such as the Private Provider Interface Agency (PPIA) model for TB control [21], executed in collaboration with the local government in Maharashtra [22]. Adopting a similar methodology to the PPIA model, PATH implemented an active case-finding program in urban Nagpur by engaging private sector informal providers. This research was funded under the STOP TB Partnership’s TB REACH initiative [23,24]. The lessons learned on basic operational requirements, software installation, and stakeholder engagement are presented in this article.

## Materials and methods

### a) Qualitative evaluation of implementation challenges

For qualitative evaluation, regular monthly meetings were conducted among the project staff of the laboratories to understand the implementation challenges and to incorporate the changes for the successful adoption of AI solutions. A semi-structured questionnaire (S1 Questionnaire) was used during meetings to initiate discussions, and the challenges related to operational restraints and acceptability were then analyzed. The adapted changes that worked for program implementation are reported in this article.

### b) Tuberculosis Diagnostic Algorithm

The active case-finding program implemented between September 2018 –March 2020 targeted the slum dwellers of Nagpur who have limited access to health services due to financial constraints and physical inaccessibility to quality health facilities. The informal providers practicing in one of the six most prevalent systems of medicine practiced in India (Ayurveda, Yoga, Naturopathy, Unani, Siddha, and Homeopathy [AYUSH]) were engaged in considering the advantages of accessibility, affordability, and a high degree of trust within the community [25–27].

**Fig 1:**
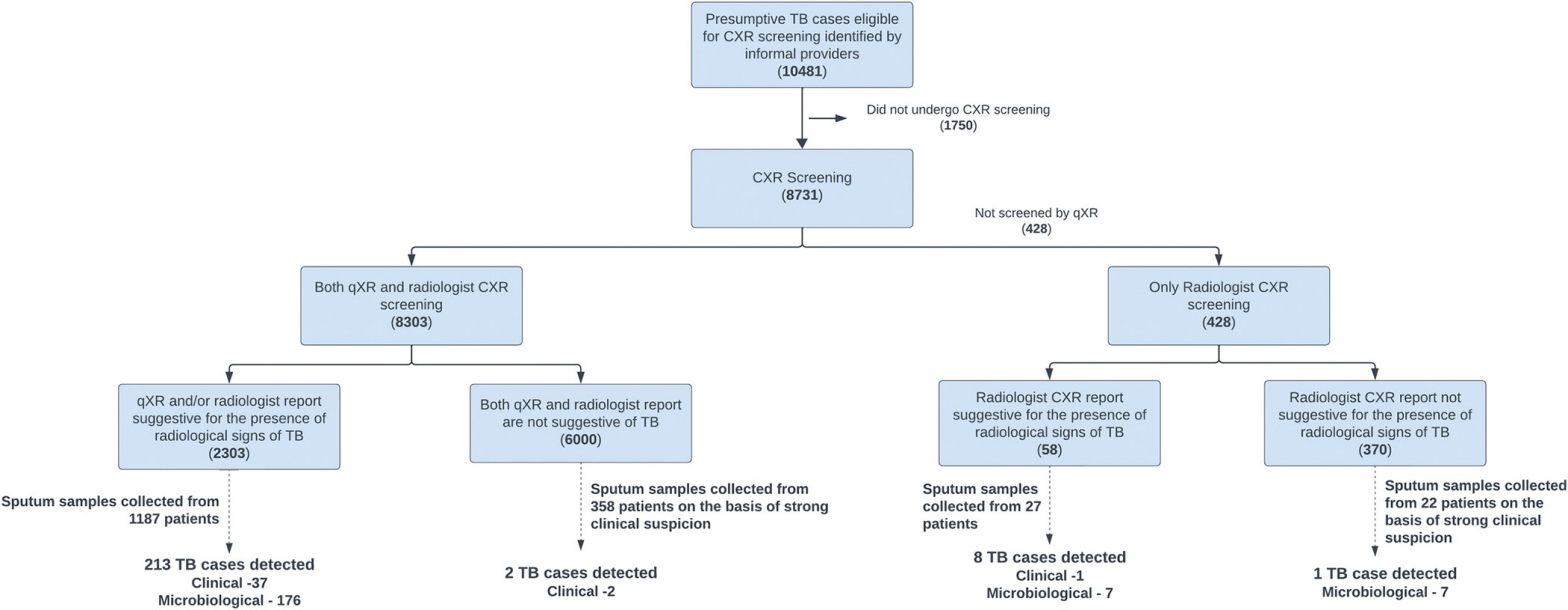
Active case finding algorithm and results from the project implementation period (Numbers in parentheses indicate absolute numbers in each category).

As part of our diagnostic algorithm (Fig 1), the informal providers screened individuals turning up in clinics for presumptive TB identification based on clinical history using a verbal screening method utilizing socio-demographic characteristics, clinical symptoms, previous history of TB, and other illnesses of the individuals. Once identified, the individuals were provided with a CXR voucher and referred to a nearby private CXR laboratory, mapped, and engaged with the TB REACH project, where CXR screening was conducted free of cost. Radiologists and the qXR AI software read all CXRs. Abnormal findings reported on qXR were reported to a healthcare worker as text messages generated using the qTrack application, also developed by Qure.ai [28]. All the individuals receiving CXR vouchers were assigned with alphanumeric case IDs. CXRs were uploaded on the cloud with these IDs, and personal identifiers were not shared for screening with the software device manufacturer (Qure.ai) or any other external stakeholders. Fig 1 illustrates the diagnostic algorithm of the active case-finding program.

Individuals identified as presumptive for TB through an abnormal CXR reading by either a radiologist or qXR were referred to a government testing facility (public sector) for microbiological testing using TrueNat assay [29]. Individuals diagnosed with TB were initiated on treatment at a nearby DOTS (Directly Observed Therapy, Short-course) center and followed up by health volunteers engaged with PATH. To avoid biases, radiologists were blinded to the AI results. A decision threshold score of 0.5 was used as a threshold of abnormality to detect TB in CXR, and this was the manufacturer-recommended default threshold. The diagnostic accuracy and validation of qXR have been previously reported in research studies [13,14,16,17]. Hence, no performance evaluation of the algorithm, sample size estimation, or statistical analysis was performed as a part of this project.

We also compared the number of TB notifications during the project implementation period to the historical baseline data from the same geographical region to investigate the increase in TB notifications. We used the NTEP data management system for data sourcing. Descriptive results from this comparison are also reported.

### Ethical considerations

PATH received approval from the Nagpur City TB office to implement their TB REACH project as part of programmatic services; thus, no additional ethical approval was required. All information was anonymized, and only aggregate data were used in the analyses. Lessons learned were gathered during staff meetings where PATH staff voluntarily provided information within their professional capacity and directly related to the programmatic implementation of the active case-finding strategy.

## Results & Discussion: Lessons learned from implementation

The project implementation was the first programmatic roll-out of any AI software device within the private CXR facilities in Nagpur, resulting in subsequent learnings. The lessons learned in this qualitative evaluation focused on two main areas: operational feasibility and acceptability. Operational feasibility was further subcategorized into two categories: 1) basic set-up requirements for AI software installation and 2) digital imaging and communication in medicine (DICOM) image viewing software.

### 1. Operational feasibility

#### 1.1. Basic set-up requirements: Internet, LAN, and administrative rights

To read CXRs, qXR script installation is required, thus requiring, at a minimum, 1) a local area network (LAN), 2) a static internet protocol (IP) address, 3) transfer of administrative rights allowing for remote access, and 4) operating system configuration. For labs using a 2-system set-up, LAN was required to ensure digital CXR copies were transferred successfully from one computer to the other for screening. A static IP (internet protocol) address, transfer of administration rights, and remote access were required to ensure that the Qure.ai team could adequately install the application, ensure maintenance throughout the project duration, and assist with any troubleshooting. Further, as the software being installed at the respective site was done remotely and via internet access, a transfer of administrative rights was required. However, physical installation by a member of the Qure.ai team would also be an option.

For program implementation purposes, lab owners were hesitant to install the qXR script due to the transfer of administrative rights. For this reason, out of 8 labs engaged in this program, six labs used the 2-system set up to ensure that their lab computer was independent of that required for qXR reading and conversion of DICOM file formats. With the help of the Qure.ai team, a script was designed to ensure the proper transfer of files from one computer to another without manual intervention, and educational materials were distributed to all lab owners to ensure confidence in the system.

While this was not a substantial challenge, the operating system configuration may be an aspect to consider when using qXR or AI interpretation algorithms. Other system requirements include that the computer used for software installation must have a minimum of 10 GB of free space. It should also have updated software of Windows 7 SP2, or MacOS Catalina, or Ubuntu 14, or later versions.

#### 1.2. Digital imaging and communications in medicine image viewing software & qXR software installation

**Fig 2:**
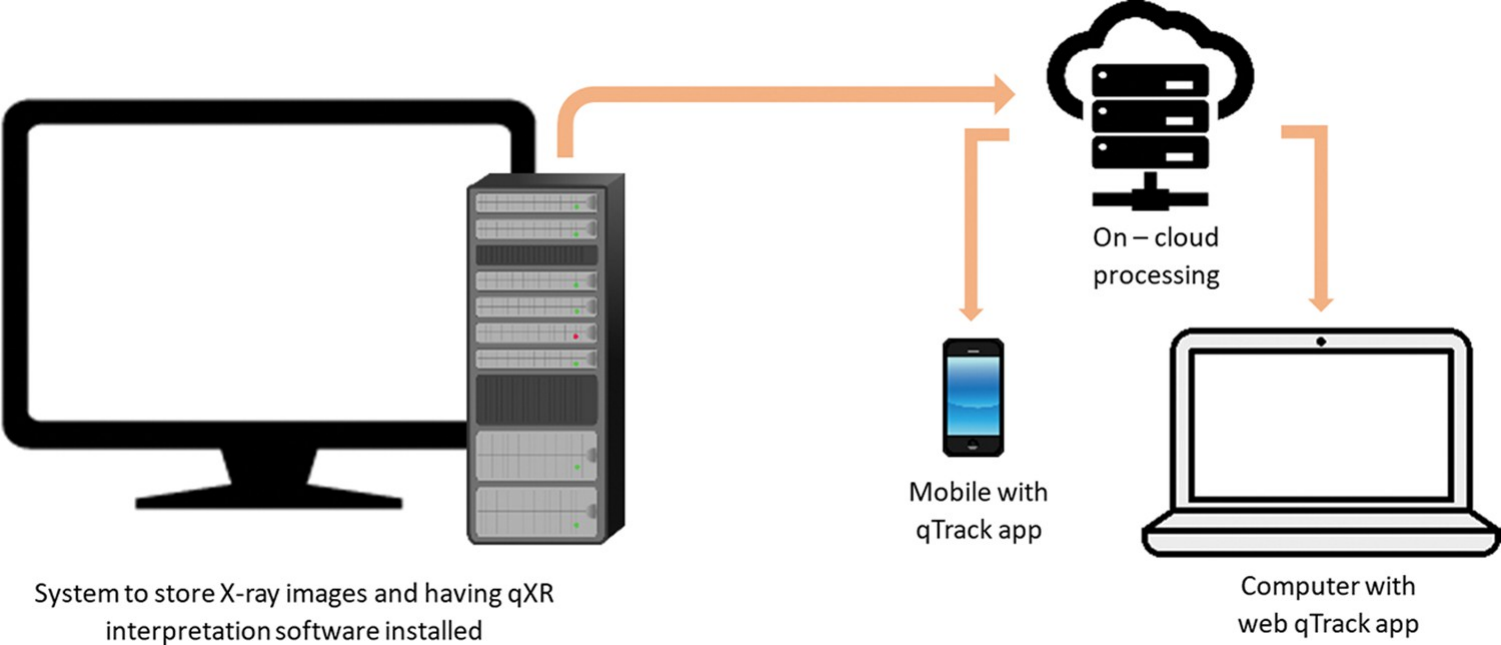
One-system set up for qXR reading.

**Fig 3:**
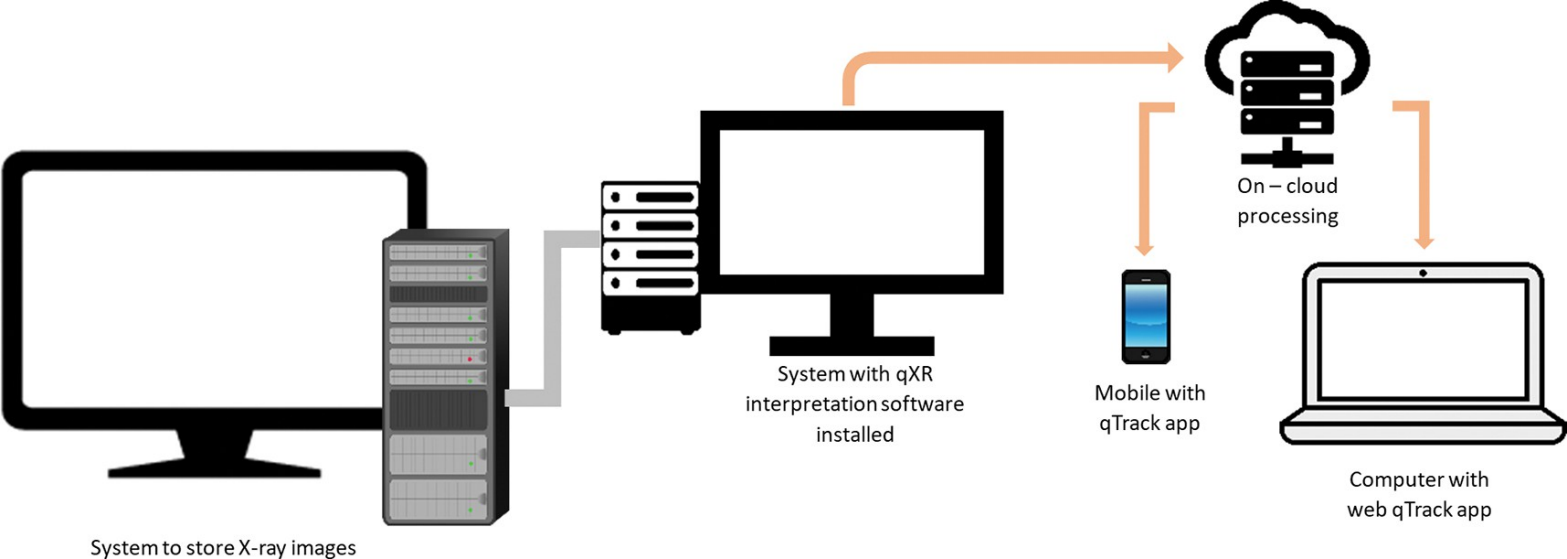
Two-system set up for qXR reading.

Digital Imaging and Communications in Medicine (DICOM) is the standard for communicating and storing medical images between computers [30]. Installation of a DICOM viewer application is required on CXR lab computers to view images taken by the digital CXR machine. This application can be installed using two methods: 1) 1-system set up: qXR interpretation software installed on the same computer used to store CXR images, or 2) 2-system set up: one computer to store CXR images and a second computer contains qXR interpretation software. As shown in Fig 2, the 1-system setup enabled immediate transfer of CXR images to the cloud server for interpretation. Results were subsequently reported to PATH through the designated health care worker for subsequent linkage for diagnosis. Fig 3 illustrates the 2-system setup, which enables the transfer of CXR images from one computer to another and the next transfer to the cloud server for interpretation. Reporting of results is like that of the 1-system setup. DICOM image viewing software is needed for qXR interpretation in both forms.

Certain labs in this implementation program had digital CXR file formats, which were automatically saved into formats other than DICOM. As this is not the standard file format for X-ray machines, the images from these labs could not be read by the qXR system. Through collective discussions among all partners in the project, the qXR application was updated using requests from the project staff and subsequently installed on all computers. This permitted reading digital CXR file formats in all labs, irrespective of their automatic file formats.

### 2. Acceptability

#### 2.1. Stakeholder engagement: Radiology associations

In addition to the software requirements for installing qXR, stakeholder buy-in is also critical. This case involved a vital stakeholder group, the Radiology Association in Nagpur, and due to their hesitancy in adopting the technology, the local Radiology Association initially did not approve the installation of qXR in CXR laboratories of Nagpur. The initial reluctance towards adopting a new technology like AI was mainly due to a lack of awareness of AI and its regulatory clearance in the Indian context. Thus, over 25 private CXR facilities associated with the Radiology Association could not be included in this implementation program. However, this was mitigated by following good practice and ensuring transparency with the Radiology Association through regular meetings with their project staff. After multiple discussions and sharing of study-related documents and supporting documents on public sector AI initiatives, the local Radiology Association agreed to install qXR in CXR laboratories. Still, the data from these 25 CXR laboratories were not included in this program due to project timeline constraints. But keeping this experience in mind, early engagement with the Radiology Association is suggested for future implementations to ensure stakeholder buy-in prior to implementation. Furthermore, engaging with local government authorities was recommended as their presence may also be helpful in meetings with the Radiology Association and ensuring the necessary infrastructure to be present at CXR facilities. Additionally, providing periodical education on the novel product features or qualities and presenting how AI can act as a complementary test or a secondary reader in the TB diagnostic algorithm is also significant. This will also help avoid misleading conceptions that consider AI a replacement for radiologists.

This collective evaluation did not further assess the initial hesitancy of the Radiology Association in accepting AI-based solutions, as acceptance of new technologies can be influenced by many factors such as perceived benefits and ease of use of the technology; however, equally important is to assess perceived risks of using new technology [31]. While AI offers solutions to health care delivery, it potentially can be perceived as replacing human expertise. Further insight into reasons for hesitancy in accepting the tools through qualitative interviewing could help provide the insight needed to identify solutions to improve acceptance.

### Supplementary outcome

In total, 33 private CXR laboratories were mapped in Nagpur, of which eight (24.2%) were engaged in the implementation as they met the basic requirements such as internet, local area network, etc., and agreement for qXR installation indicated by Qure.ai. The engaged CXR laboratories conducted between 7 to 4,125 CXRs per month. This allows for the generalizability of this program implementation, as the highly productive labs producing over 4000 CXRs per month and others with a very minimal number of CXRs per month are involved.

After the project start-up period of 3 months (September 2018 to November 2018), the actual implementation took place between December 2018 and February 2020, where informal providers identified 10,481 presumptive TB individuals through verbal screening. It was suggested to undergo CXR screening by giving CXR vouchers for testing (Fig 1). 8,731 (83.3%) underwent CXR screening, and the remaining 1,750 (16.7%) patients did not take a CXR. The project compensated CXR cost through a voucher that could be redeemed. The cost barrier for the patient is overcome, which could be a reason for this high uptake of vouchers. 8,303 (95.1% of 8,731) of the CXRs were processed by qXR and were interpreted by a radiologist. The other 428 CXRs (4.9%) underwent only radiologist-based screening for technical reasons such as internet outages.

Out of the 8,303 patients who received both qXR and radiologist interpretation, 2,303 (27.7%) were found to have radiological signs of TB by either a radiologist or qXR. These 2,303 patients were advised to use a sputum sample for doing the TrueNat test. Ultimately, sputum samples were collected from 1,187 (51.5%) patients in this group and 213 patients were confirmed with TB (37 clinical and 176 microbiological). The majority of these 213 patients (n = 176, 82.6%) had been flagged earlier by both radiologist and qXR during screening; 8 (3.7%) patients were flagged by radiologist alone, and 29 (13.7%) cases were flagged by qXR alone. Thus, approximately a 15.8% increase in TB case detection could be attributed to qXR. Nevertheless, they all underwent radiologist screening, and 9 (2.1%) TB cases were detected in this group.

6,000 patients had CXRs, which were not suggestive of the presence of radiological signs of TB by either radiologist or qXR. However, due to strong clinical suspicion of TB, 358 of those patients were advised to undergo microbiological confirmation, and all of them were found to be microbiologically negative for TB, but 2 of them were clinically confirmed for TB. Clinically confirmed patients were microbiologically negative but were still started on anti-tuberculosis treatment based on the clinician’s discretion. This clinical confirmation was based on personal and family clinical history, CXR findings, and physical/biochemical examination of the patient.

While comparing the number of TB notifications during the project implementation period (January 2019 to March 2020) with historical baseline data (data from October 2017 to December 2018) from the same geographic region, we found an increase of 20 percent in notifications in the implementation period. There was also a significant increase of 50 percent in bacteriological (microbiological) notifications compared to the baseline data notifications in the implementation area (Table 1). Some proportion of this increase can be attributed to the implementation of this active case-finding strategy using informal providers and the deployment of an AI solution (qXR), as more than 80 percent of the project notifications were bacteriologically confirmed. The NTEP algorithm was symptom-screening and then sputum smear examination at the time of implementation. Since we used CXR as a filter to triage further, those flagged based on CXR findings were tested with a molecular test to confirm TB. The higher proportion of bacteriologically confirmed TB cases during the project implementation could be due to the availability of better tools for screening and confirmation of TB.

**Table 1:** Tuberculosis notifications during project implementation and baseline period.

Impact on Case Notifications	Notifications		Baseline period notifications minus implementation period	Percentage change of notifications from baseline to implementation period
	Baseline Period	Implementation Period		
**Bacteriological notifications**	682	1,020	338	50%
**All forms notifications**	1,508	1,803	295	20%

### Recommendations and Conclusion

Through well-established relationships with all the stakeholders, such as the project implementation team, technology partners, local CXR laboratories, and radiologists, we were able to mitigate various challenges during this AI implementation. Thus far, multiple studies have evaluated the diagnostic accuracy of qXR and other similar AI software devices for TB screening against human readers. However, limited literature shows methods of implementation of AI, especially in resource-limited environments. AI is well-equipped to reduce the workload on CXR laboratories and provide accurate results. The effective use of AI for future active case-finding implementations can be achieved with the following four recommendations:

1. Consistent communication with stakeholders and sensitization on AI, especially the radiology association, to ensure seamless installation, reading, and implementation of AI within their settings.
2. Communicating the possibilities of a two-system setup to avoid reluctance from CXR laboratory owners on breach of administrative rights.
3. Ensuring robust internet connectivity for AI screening–internet dongles or data cards are not optimal for such use. However, AI vendors may have additional capabilities that enable the offline use of the devices, which is helpful in settings with no internet connectivity.
4. Allotting a significant amount of time before implementation for system installation and deployment.

AI interpretation solutions, such as qXR, can potentially improve TB care and services. However, the successful implementation of new digital health innovations requires consideration of feasibility, acceptability, ease of use, and user experience. Future studies evaluating AI tools are encouraged to incorporate such qualitative aspects into their study design to facilitate implementation and enable modifications as required.

## Supporting information

S1 Questionnaire Semi-structured questionnaire. (DOCX)
