# Supplementary material for: Implementing a chest X-ray artificial intelligence tool to enhance tuberculosis screening in India: Lessons learned
Source: PLOS Digit Health. 2023 Dec 7;2(12):e0000404. doi: 10.1371/journal.pdig.0000404 (PMC10703224; doi:10.1371/journal.pdig.0000404)
Supplement: S1 Questionnaire — (DOCX) [file pdig.0000404.s001.docx]

**REASONS FOR LIMITED REFERRAL: AN INFORMAL PROVIDER’S PERSPECTIVE**

**Context:**

TB REACH Wave 6 intervention implemented by PATH Mumbai, in conjunction with implementation partner DISHA foundation, has been focusing on informal private provider engagement. The intervention engages informal private providers in urban Nagpur by encouraging them to refer symptomatic TB patients to CXR labs. These laboratories are equipped with Qure’ai, an automated CXR reading system developed in Mumbai, India. The informal private providers provide CXR referral slips to the symptomatic TB patients to allow free-CXR screening. Based on initial intervention results, the dropout rate between referrals and CXR screening is 21%. As this is a pilot intervention, the team aims to understand the reasons behind the lack of referrals in order to improve the intervention from the perspective of the informal private providers.

**Research objective:**

To understand and identify the reasons for limited referrals by informal private providers in urban Nagpur, Maharashtra.

**Methods:**

- Study design:

The study will follow a qualitative cross-sectional study design.

- Target population:

The target population will be the engaged informal private providers in Nagpur, Maharashtra. These private providers must have signed an MOU to participate in the TB REACH Wave 6 intervention.

- Sampling strategy:

There are 430 providers who have agreed to engage with the TB REACH intervention and have signed MOU’s with PATH. These providers have been mapped using Kobotoolbox. We will use a purposive sampling strategy. The sampling strategy has been separated into 3 parts (see appendix). Firstly, the TU’s with the highest number of informal private providers will be selected, among those TU’s informal private providers will be classified based on their degree and then 20% from each degree category will be chosen to be eligible for this study. Lastly, providers will be classified into 3 broad categories; a) no registrations, b) 1-5 presumptive TB patient registrations and 3) greater than 5 presumptive TB patient registrations. Informal private providers registrations will be classified based on the last three months (Q3 2019). Based on the sampling strategy, a sample size of 40 providers will be sufficient. Table 1 presents the breakdown of providers, based on classification.

| **Provider Category** | **TB Unit - DTC** | **20% sample** | **TB Unit - GAC** | **20% Sample** |
| --- | --- | --- | --- | --- |
| AYURVEDA | 63 | 13 | 52 | 10 |
| Homeopathic | 23 | 5 | 31 | 8 |
| UNANI | 20 | 4 | 2 |  |
| Grand Total | 108 | 22 | 85 | 18 |

**Table 1: Category of providers by TU and proposed sample size**

*DTC: District TB Center GAC: Government Ayurvedic Center

- Data collection method – interviews:

Informal semi-structured interviews will be conducted by field coordinators with the informal private providers. Interviews will be conducted in Hindi and field coordinators will take notes on the interview sheet throughout the interview. They will be conducted at a time and location most convenient to the providers.

The interview guide (see appendix) contains a series of sociodemographic information and three open-ended questions. Prior to conducting the interviews, the field coordinators will be trained by PATH research staff on interview and probing techniques. Written consent (see appendix) will be taken from all informal private providers prior to conducting the interview. The data collected from the interviews will be referred as “notes from the field”.

- Data analysis:

The paper-based interview notes will be transcribed onto Excel by two members of the PATH team. The notes will subsequently be analyzed using thematic analysis. The team will identify major and minor themes explaining the reasons behind limited referrals. Microsoft Excel will be used for all analyses, including thematic analysis.

**Dissemination output:** An internal report for PATH Mumbai’s core team and the TB REACH Secretariat. This internal report will serve to improve the TB REACH intervention.

**APPENDIX A: INTERVIEW GUIDE**

**Socio demographic information:**

1. Facility type
2. Provider gender
   1. Male
   2. Female
   3. NR
3. Age:
   1. 18-23
   2. 24-30
   3. 31-40
   4. 41-50
   5. 50+
   6. NR
4. Qualification:
   1. BAMS
   2. BHMS
   3. BUMS
   4. LCEH
   5. Other
   6. NR
5. OPD load per day:
6. Coughing patients screened per day
7. Types of services provided:
   1. Screening
   2. Referral
   3. Diagnosis
   4. Treatment adherence support
   5. Other, please specify:
   6. NR

**Interview questions:**

1. How do you screen your coughing patients?
2. Do you screen presumptive TB patients?
   1. If no, where do the patients go?
3. Do you refer presumptive TB patients to CXR laboratories?
   1. Yes
   2. No
   3. Other
   4. NR
4. What are the reasons for referring (or not) the symptomatic patients to CXR laboratories?
   1. Do you have an agreement with another laboratory? If yes, which are these labs
   2. Do you use the services offered in your own laboratory
   3. Do you feel that screening isn’t required, and just diagnosis right away helps
   4. What is your opinion on screening? Do you feel it is required, why or why not?
   5. Are there any other reasons for non-referral?
5. Do you provide free CXR vouchers to your presumptive TB patients?
   1. If yes, how many vouchers do you provide in a week?
      1. Do these patients return to your clinic after diagnosis as well?
   2. If no, are there any barriers in providing vouchers. Also how do you refer the patients for CXR?
   3. What do you like about the free voucher system?
   4. What do you dislike about the free voucher system?
6. Do you follow-up with your TB patients to see if they utilized the CXR voucher and received a chest x-ray report?
7. Do you have any suggestions for the field team to ensure patients utilize the CXR voucher for screening?
8. What has been your experience engaging with this private sector TB control intervention
9. Do you treat TB patients or refer them to public sector or private sector for treatment?
10. Do you perceive any barriers for female patients to visit the clinic for health issues and also for TB diagnosis? Yes/No
    1. If yes, why?

**APPENDIX C: SAMPLING**

The sample size calculation was done in three stages. The first stage involved identifying the TU’s with the highest number of providers. TU District TB Centre and Government Ayurvedic College were identified as those with the highest number of informal private providers. These two TU’s were chosen, to allow for responses from each different provider category.

Table: Distribution of providers by TB units

| TU Name | AYURVEDA | Homeopathic | UNANI | Others | Total |
| --- | --- | --- | --- | --- | --- |
| CHAKOLE | 49 | 30 | 1 |  | 80 |
| Dr. Ambedkar | 14 | 6 | 2 |  | 22 |
| DTC | 63 | 23 | 20 | 3 | 109 |
| GAC | 52 | 31 | 2 |  | 85 |
| IGGMC | 18 | 14 | 17 |  | 49 |
| Mahal | 3 | 3 |  |  | 6 |
| SADAR_DIA_CENTRE |  | 1 | 1 |  | 2 |
| SHANTI NAGAR | 45 | 23 | 10 |  | 78 |
| Grand Total | 244 | 131 | 53 | 3 | 431 |

Secondly, the informal private providers were categorized based on their degree and 20% of the providers from each category were selected on pro-rata basis.

Table: Category of providers by TU and proposed sample size

| Provider Category | DTC | 20% sample | GAC | 20% Sample |
| --- | --- | --- | --- | --- |
| AYURVEDA | 63 | 13 | 52 | 10 |
| Homeopathic | 23 | 5 | 31 | 8 |
| UNANI | 20 | 4 | 2 |  |
| Grand Total | 108 | 22 | 85 | 18 |

Finally, each individual degree will be further classified based on the number of registrations in the last three months (Q3 2019). The classifications will be as follows; a) no registrations, b) 1-5 registrations and 3) more than 5 patients registered. Sampling frame each categories will be computed by using the patient registration data and will be selected randomly.

Table: Sample size for each TB unit and provider category

| Degree | Registration | DTC | Sample | GAC | Sample |
| --- | --- | --- | --- | --- | --- |
| AYURVEDA | 0 | 25 | 5 | 21 | 4 |
|  | 1-5 | 26 | 5 | 18 | 3 |
|  | >5 | 12 | 3 | 13 | 3 |
|  | Total | 63 | 13 | 52 | 10 |
| Homeopathic | 0 | 11 | 2 | 19 | 4 |
|  | 1-5 | 5 | 1 | 6 | 2 |
|  | >5 | 7 | 2 | 6 | 2 |
|  | Total | 23 | 5 | 31 | 8 |
| UNANI | 0 | 6 | 1 |  |  |
|  | 1-5 | 10 | 2 | 1 |  |
|  | >5 | 4 | 1 | 1 |  |
|  | Total | 20 | 4 | 2 |  |

**APPENDIX B: CONSENT FORM**

**Title:** The reasons for limited referrals – an informal provider’s perspective

**Purpose:** To understand the reasons for limited referrals by informal providers in urban Nagpur, Maharashtra.

**Process of interview:**

PATH Mumbai and Disha foundation joint intervention to improve TB case detection among informal private providers in urban Nagpur, India. The team had contacted you earlier in the year to request your engagement with the intervention. The organizations have noticed a low referral rate from informal private providers and would like to further understand why through informal interviews.

The informal semi-structured interview will take place in a location and time that is most convenient to you. The field coordinator will ask you a set of sociodemographic questions followed by 3 open-ended questions. If you require any clarification or have any query, please do not hesitate to ask the field coordinator. This interview will take 30-45 minutes.

**Risks and confidentiality:**

There will be no risks posed to you while conducting this interview. All information provided will be anonymous and confidential. Only the study team will have access to the paper based as well as digital records of the data collected. Further, the data will be kept at the PATH Mumbai office in a locked cabinet and password protected laptop.

**Compensation:**

You will not receive any supplementary compensation for participating in this research study.

**Voluntary participation:**

If you choose to leave the study at any point in the interview, you will be allowed to leave with no questions asked. If you do not want to answer a question, you can say so as well.

**Contact information:**

Vaishnavi Jondhale, TB REACH team lead

PATH Mumbai, Mumbai, Maharashtra

Email: [vjondhale@path.org](mailto:vjondhale@path.org)

Do you consent to participate in this study?

- Yes
- No

Signature: ___________________

Date
